# P-475. Risk Factors for Nosocomial Infection (NI) in Pediatric ECMO Patients at a Tertiary Health Care Center: A 15-year Retrospective Review

**DOI:** 10.1093/ofid/ofaf695.690

**Published:** 2026-01-11

**Authors:** Ali Abolhassani, Caitlyn L Margol, Muhammad Ashraf, Alleigh Wettstein, Shawn Doss, Luke Guy, Grace Thayer, Karim Jandali, Malek Moumne, Pinkalkumar Patel, Laura L Hampton, Ingrid Camelo

**Affiliations:** Medical College of Georgia at Augusta University, Evans, GA; Medical College of Georgia at Augusta University, Evans, GA; Wellstar Medical College of Georgia Health Medical Center, Augusta, Georgia; Medical College of Georgia at Augusta University, Evans, GA; Medical College of Georgia at Augusta University, Evans, GA; Medical College of Georgia at Augusta University, Evans, GA; Medical College of Georgia at Augusta University, Evans, GA; Medical College of Georgia at Augusta University, Evans, GA; Medical College of Georgia at Augusta University, Evans, GA; Wellstar Medical College of Georgia Health Medical Center, Augusta, Georgia; Medical College of Georgia at Augusta University, Evans, GA; The Johns Hopkins University School of Medicine, Baltimore, Maryland

## Abstract

**Background:**

Extracorporeal membrane oxygenation (ECMO) has been shown to reduce mortality in critically ill pediatric patients but carries a high risk for significant complications, including infection. This study reviews 15 years of ECMO data to identify risk factors for nosocomial infection.

**Table 1:**
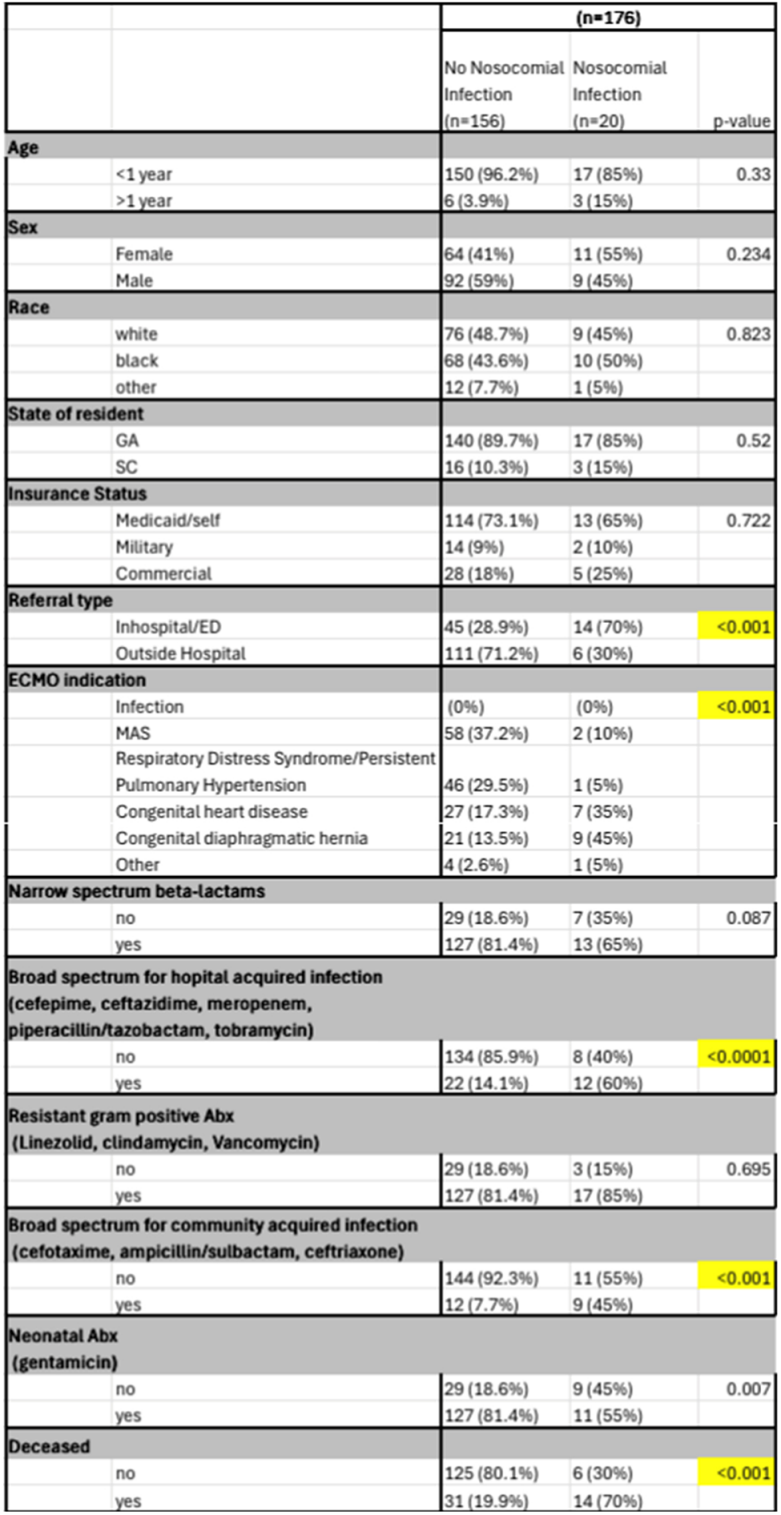
Descriptive Analysis of Patient Demographics

**Table 2:**
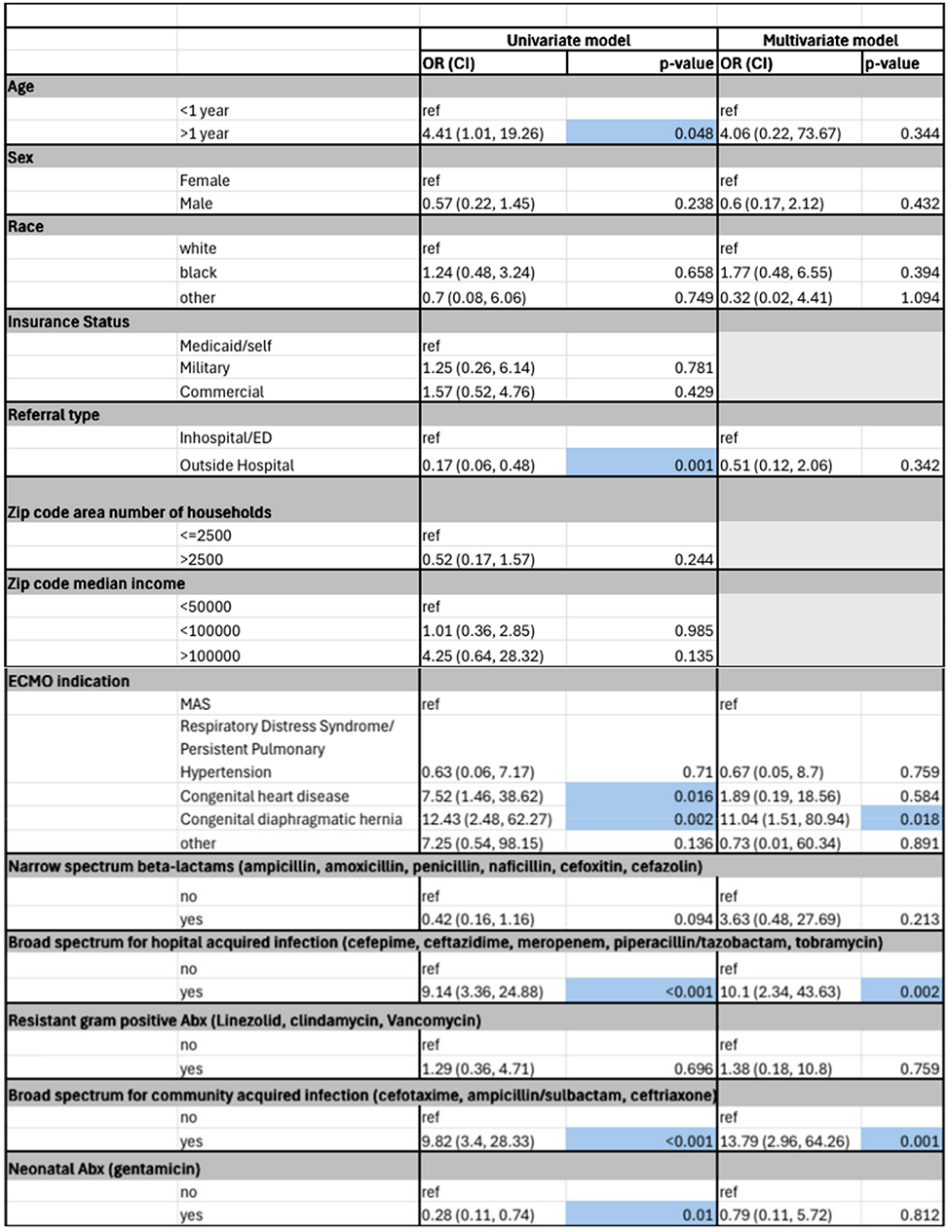
Univariate and multivariate logistic regression to predict Peri-ECMO nosocomial infection in pediatric ECMO patients without infection (n=176)

**Methods:**

Retrospective chart review of pediatric patients on ECMO admitted to a single tertiary care hospital was conducted. ECMO-related nosocomial infections (NI) were defined as a positive viral, bacterial, or fungal pathogen detected from any source between 48-hours post cannulation and 24-hours post decannulation, as an adaptation of Centers for Disease Control and Prevention (CDC) NI infection criteria. Antimicrobials were categorized by the National Healthcare Safety Network criteria. Logistic regression assessed associations between risk factors and NI. Indications for ECMO cannulation were meconium aspiration syndrome (MAS), respiratory distress syndrome/persistent pulmonary hypertension, congenital heart disease (CHD), and congenital diaphragmatic hernia (CDH).

**Results:**

Of 176 ECMO patients canulated for noninfectious causes, 20 met criteria for NI (11.3%). A multivariate logistic model showed increased odds for NI among patients with CDH (OR=12.4 (95% CI 2.4-62.2) p=0.002) compared to those cannulated for MAS. Exposure to broad spectrum antimicrobials for community-acquired infections (OR=13.7 (95% CI 2.96-64.2) p ≤0.001) and exposure to broad-spectrum antimicrobials for hospital-acquired infections (OR=10.1 95% CI 2.3-43.6) p ≤0.001) were significantly associated with development of NI related to ECMO.

**Conclusion:**

CDH-related ECMO cannulation carries over 11 times higher odds of developing NI in the multivariate model. There are currently not well established ECMO-directed infection control protocols in pediatrics. Early CDH identification should trigger tighter line-care infection control bundles, focused surveillance cultures, and diagnosis-guided antibiotic plans while helping teams set realistic expectations for families. These data support multicenter validation and refinement of ECMO practices to reduce infection burden and improve survival in this high-risk group.

**Disclosures:**

All Authors: No reported disclosures

